# A Question for Women's Health: Chemicals in Feminine Hygiene Products and Personal Lubricants

**DOI:** 10.1289/ehp.122-A70

**Published:** 2014-03-01

**Authors:** Wendee Nicole

**Affiliations:** **Wendee Nicole** was awarded the inaugural Mongabay Prize for Environmental Reporting in 2013. She writes for *Discover*, *Scientific American*, *National Wildlife*, and other magazines.

Vaginal research got a desperately needed boost at the National Institutes of Health (NIH) in 1992. That’s when Penny Hitchcock took over the Sexually Transmitted Diseases Branch at the National Institute of Allergy and Infectious Diseases and Nancy Alexander became chief of the Contraceptive Development Branch in the NIH Center for Population Research—posts previously held by men. “Those two got together and discovered NIH had no programs for vaginal research,” says Richard Cone, a Johns Hopkins biophysics professor. Cone had begun developing vaginal contraceptives that would protect against sexually transmitted infections (STIs) in 1980, and until then, struggled to get funding.

**Figure:**
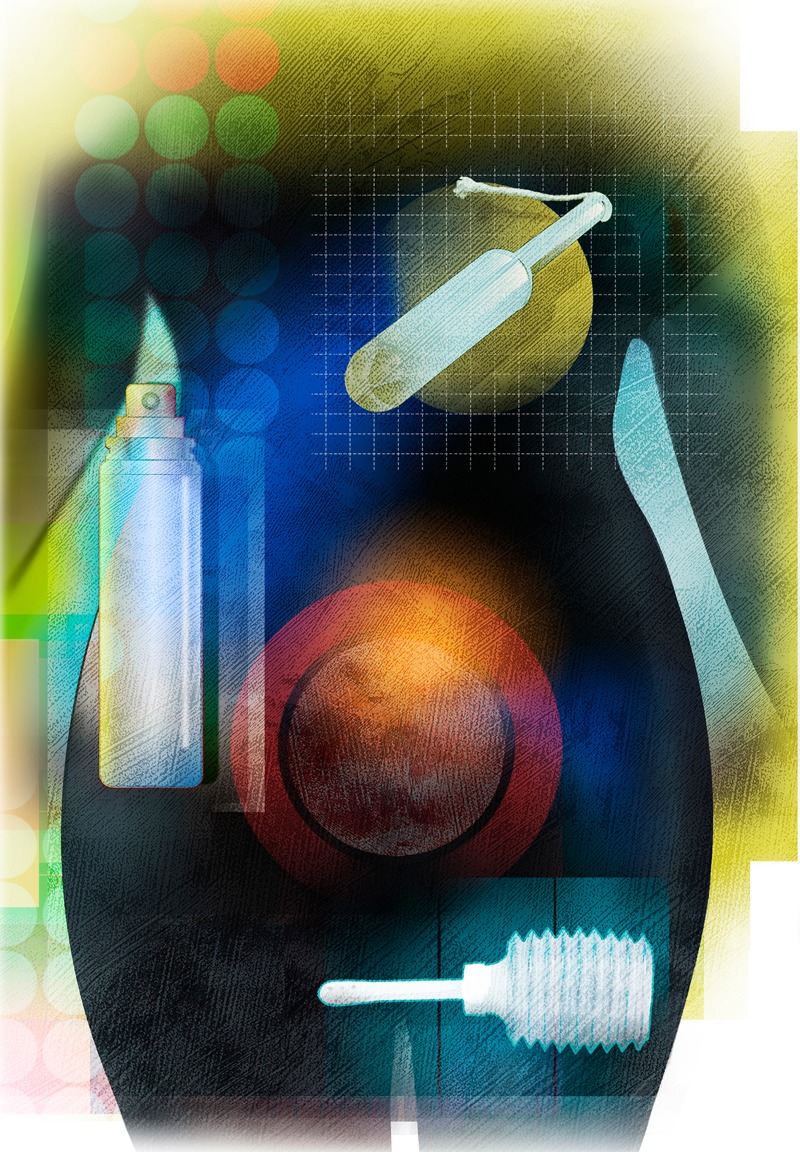
Mucous membranes in the vagina and vulva rapidly absorb chemicals without metabolizing them. But until recently scant research existed on how chemicals in feminine hygiene products and personal lubricants may affect women’s health. © Roy Scott

Hitchcock and Alexander soon initiated research programs on vaginal physiology, immunology, and microbicides, eventually funding Cone’s work. These new programs led to groundbreaking discoveries in animals and humans that certain chemicals—including glycerin (glycerol), a common base for personal lubricants—can damage or irritate vaginal^1^ and rectal^2^ epithelial cells, potentially increasing the transmission of STIs such as herpes and human immunodeficiency virus.

When it comes to reproductive health, research on contraceptives and STIs continues to garner interest worldwide. But a related area—chemical exposures from feminine hygiene products and personal lubricants—has received much less attention. In the United States alone, women spend well over $2 billion per year on feminine hygiene products,^3^ including tampons, pads, feminine washes, sprays, powders, and personal wipes. But until recently, scant research existed on how chemicals in these products may affect women’s health. As scattered findings emerge, several scientists and interest groups are calling for more research to fill in the gaps.^4^^,^^5^

A recent report by the nonprofit Women’s Voices for the Earth (WVE) points out that feminine hygiene products may use ingredients that are known or suspected endocrine-disrupting chemicals (EDCs), carcinogens, or allergens. And while nearly all women use tampons and sanitary pads, black and Latina women use douches, wipes, powders, and deodorizers more often than women of other races, putting them at greater risk of potential chemical exposures.^4^

“It’s a little bit edgy. People have been shocked, saying things like ‘I’ve never really thought about [the vagina] being an important internal link to your body,’” says Alexandra Scranton, director of science and research for WVE. “Although it is well known that the vaginal ecosystem is more sensitive and more absorbent than typical skin, there is surprisingly little research out there on feminine care products.”

**Figure:**
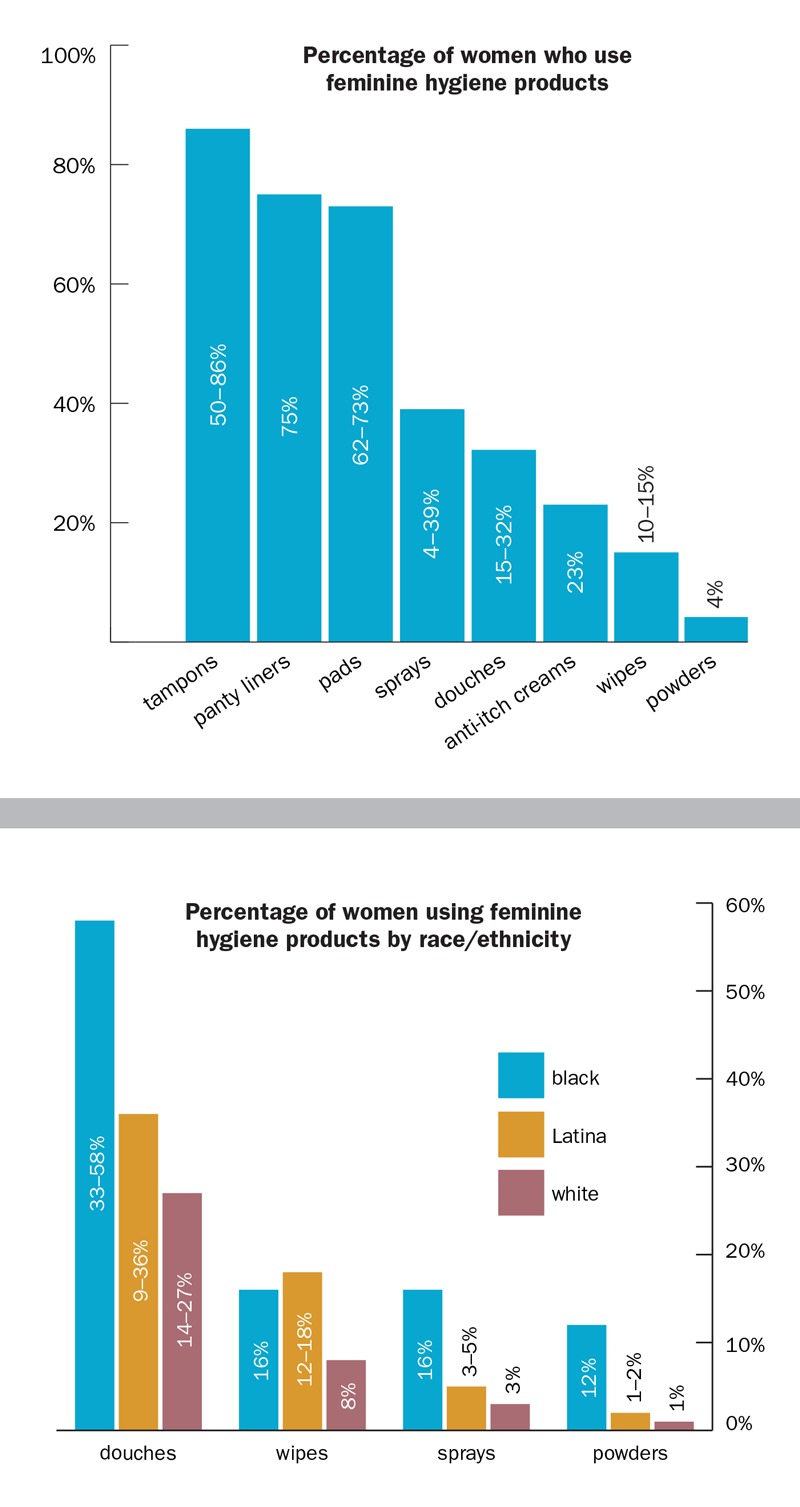
Surveys conducted in the 1990s–2000s gave a sense not only of how commonly some products are used but also how widely use can vary across racial/ethnic groups. Adapted from Scranton (2013)^4^

## The Vaginal Route of Exposure

Female sex organs evolved to be self-cleaning.^6^ The vaginal canal is richly endowed with blood vessels and produces mucus that protects against and washes away harmful microorganisms.^7^ As a mucous membrane, the vagina is capable of secreting and absorbing fluids at a higher rate than skin, as are some of the external portions of the vulva, including the clitoris, clitoral hood, labia minora, and urethra.^7^^,^^8^^,^^9^

“Most of the vagina is covered with multiple layers of dead and dying cells that do a lot to protect it against infection, but [this] is nowhere near the thick leathery surface of our skin,” says Cone. “The vaginal epithelium … is highly water permeable in a way our skin is not.”

Because mucous membranes in the vagina and vulva rapidly absorb chemicals without metabolizing them, researchers have even explored the possibility of delivering drugs vaginally.^10^ One study found that vaginal application of estradiol, a synthetic estrogen, resulted in blood serum levels 10 times higher than those following oral dosing.^11^ But while rapid absorption works well when a patient needs a drug delivered rapidly, it may also expose women to higher levels of chemicals from feminine hygiene products than manufacturers intend.

“The study about the enhanced absorption of estradiol was really compelling because a lot of these chemicals [found in feminine car products] can interfere with estrogen signaling,” says Ami Zota, an assistant professor of epidemiology at George Washington University. “Plausibly the same concept would extend to other [EDCs].” Zota is researching whether fragranced feminine hygiene products, in particular, add to the body burden of EDCs in women of different racial and socioeconomic groups.^12^^,^^13^^,^^14^

Among the suspected EDCs found in some feminine hygiene products are parabens,^15^ which are used as preservatives, and fragrance ingredients including diethyl phthalate^16^ and Galaxolide_®_.^17^ (Parabens are also commonly used in personal lubricants.^2^) “Chemicals from plastics may also be of potential concern, given that many of these feminine hygiene products have applicators,” Zota says.

## Infections and Irritation

Several studies have found that black and Latina women tend to use douches and feminine deodorizers more often than women of other races, and also experience higher rates of bacterial vaginosis and yeast infections.^18^^,^^19^^,^^20^^,^^21^ Douching is also more common among women of lower socioeconomic class, especially among white women.^18^

Yet the American Public Health Association and other health groups strongly recommend not douching unless specifically medically recommended.^22^^,^^23^ Research has linked the practice with increased risk of bacterial and yeast infections, pelvic inflammatory disease, cervical cancer, increased transmission of STIs, and other adverse health outcomes.^24^^,^^25^

But despite longstanding warnings over douching, nearly half the women surveyed in one 2008–2010 study had douched in the past month.^26^ A majority learned the practice from their mothers and reported douching to feel clean and fresh, or to prepare for or clean up from sex.

“With douches we come across a lot of cultural issues about how women should smell and how women should take care of their body when it’s not necessarily related to their health,” says Ryann Nickerson, communications director for the Colorado Organization for Latina Opportunity and Reproductive Rights (COLOR). “These are cultural reflections.” She says the women would never question whether their mothers were suggesting they do something unhealthy or dangerous.

COLOR leads *cafecitos*, or coffee meetings, in the Denver area for Latina women from various walks of life, including recent immigrants. “Women and particularly women of color are not always receiving the information they need,” says Nickerson. “Individuals are becoming more aware of chemicals in shampoo, face wash, and other personal hygiene products, but when you talk about feminine hygiene products … it’s a shock.”

Vaginal yeast infections and bacterial vaginosis are common among women, and many treat suspected infections with over-the-counter medications, including imidazole antifungals, anti-itch creams, and a variety of homeopathic remedies.^27^ But without medical training, women aren’t always able to effectively diagnose themselves. One study showed that successful self-diagnosis occurred among just 34.5% of untrained women who had ever been diagnosed with a prior yeast infection and 11% of women who had not.^28^ This can lead to unnecessary application of over-the-counter treatments,^29^ potentially setting the stage for overgrowth of azole-resistant yeast species,^30^ among other possible problems.

Common issues from the use of various feminine hygiene products are allergic reactions and irritation; a number of chemicals that are otherwise relatively safe can elicit these reactions in sensitive individuals.^31^ In what Scranton considers a positive feedback loop, women may turn to over-the-counter products for relief of itching, when those products could be exacerbating the problem.

“One of the common ingredients in over-the-counter anti-itch drugs is benzocaine, which has a numbing effect so it temporarily relieves itching,” says Scranton. “[But] in studies^31^ it is one of the top triggers known to cause anogenital dermatitis [irritation and itching around the anus and genitals].” Several studies have also reported cases of contact dermatitis from sanitary pads.^32^^,^^33^^,^^34^^,^^35^

## Menstrual Products

Toxic shock syndrome (TSS) remains one of the best-known health impacts of a feminine hygiene product. Cases of TSS, which can be fatal, spiked around the same time manufacturers began using four synthetic products in high-absorbency tampons. Today, the only synthetic allowed in tampons is viscose rayon, which is often mixed with cotton. “[Viscose rayon] was the best of the bad four ingredients, three of which have been taken off the market,” says Philip Tierno, a clinical microbiology and pathology professor at New York University. A small number of TSS cases are still reported to the Centers for Disease Control and Prevention each year (reporting is not required in all states).^36^

“All [synthetic] fibers cause the production of large quantities of toxins absorbed by the vaginal mucosa, which is highly vascularized,” says Tierno, who linked TSS to a toxin produced by *Staphylococcus aureus* in the presence of synthetic-fiber tampons.^37^^,^^38^^,^^39^ Synthetic fibers are more absorbent than cotton; they concentrate menstrual proteins to a greater degree than cotton and provide “an incredibly perfect physico-chemical environment” for toxin production, Tierno says.^39^ “In the vagina, the bacteria that maintain that space are mostly anaerobic,” he says. “When you place a synthetic product in the vaginal vault, those bacteria respond to the changed physical environment.”

In decades of research, Tierno has never seen a case of TSS with exclusive use of an all-cotton tampon.^40^ The bottom line, in his opinion: “Cotton is the best possible product.” However, all tampons can cause tiny tears in the vagina, which may provide entry for other chemicals or the TSS toxin.^41^^,^^42^^,^^43^^,^^44^

Dioxins pose another concern for some consumers. These chemicals are sometimes present in trace amounts in tampons and pads as a by-product of cotton and wood pulp bleaching. A 2002 study modeled exposures to dioxins from four brands of tampons and estimated them to be insignificant compared with exposures through the food supply or other sources.^45^ A patient alert from the U.S. Food and Drug Administration (FDA) describes the risk of adverse effects from dioxins in tampons as “negligible.”^41^

No studies have tested diffusion of dioxins from tampons *in vivo*, however.^46^ According to Tierno, even minute dioxin exposures can accumulate in the body with a potential cumulative effect. “A woman uses approximately 11,400 tampons in her menstrual life,” he says. “That’s exposure to dioxins 11,400 times.”

Since 1999 Congresswoman Carolyn Maloney (D–NY) has repeatedly introduced the Robin Danielson Act—which calls for federal research on TSS, tampon use, and chemical exposures through feminine hygiene products—without success.^5^ “It was knocked down as unnecessary and a waste of money,” says Tierno, most recently in 2011. “You can bet your bottom dollar if the bulk of the representatives were female, or if these males menstruated, they would have passed it by now.”

The WVE report also raised the question of pesticides in the cotton used in feminine hygiene products. The report cites third-party testing commissioned by a consumer advocacy website, which reportedly found detectable residues of eight pesticides in one brand of tampons.^4^ “I don’t know the quality control in the lab,” says Charlene Dezzutti, an associate professor of obstetrics and gynecology at the University of Pittsburgh, “but it raises a flag, and maybe we should have a better guideline on what should be tested.”

The FDA has a lot on its plate, Dezzutti says, “but people are putting these products internally, and when you stick things inside your vagina or rectum you can have absorption.” FDA spokeswoman Morgan Liscinsky says she is unaware of any well-conducted peer-reviewed research on absorption of pesticides from tampons that would serve as the basis for regulatory decision-making.

**Figure:**
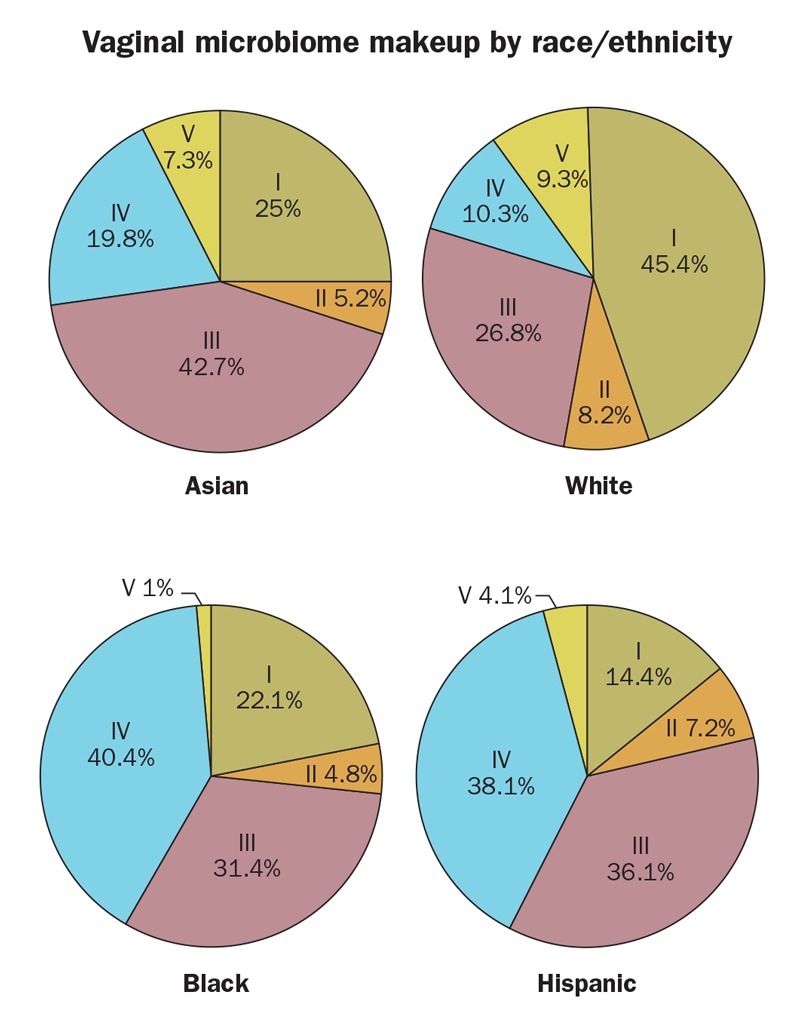
A study^55^ of the vaginal microbiomes of nearly 400 healthy women identified five major groups of microbial communities (groups I–V) that appeared in different proportions by ethnicity. Groups I, II, III, and V were dominated by *Lactobacillus* species, which are thought to play important protective roles in vaginal health. Group IV included a diversity of anaerobic species such as *Prevotella* and *Gardnerella*. Compared with white and Asian women, Hispanic and black women tended to have more group IV communities and higher vaginal pH values. The authors suggest that genetics and hygiene behaviors are just two factors that could account for the differences in microbiomes between ethnic groups. *Reproduced with permission from* Proceedings of the National Academy of Sciences

## Lubricants and STI Transmission

Like Cone, Dezzutti studies how microbicides might prevent STI transmission, and during the course of her work she discovered many personal lubricants damage human vaginal cells.^47^ “A lot of the aqueous-based lubricants are hyperosmolar, [which means] they tend to pull water out of your cells, and that causes the cells to shrink and shrivel,” she explains. “When we looked at human tissue, the cervical epithelium fractured off, and the rectal mucosa came off as well.”

Lubricants have a range of osmolalities (i.e., concentrations of solutes).^48^ Dezzutti found that lubricants with osmolalities close to extracellular body fluid had the least effect on cell viability.^47^ “The products we found safest in our paper were silicone-based lubricants … and the lubricant for the Female Condom_®_,” says Dezzutti. “Two water-based ones we found that were safe were Pre-seed [Fertility-Friendly lubricant] and Good Clean Love.” Dezzutti suspects that vaginal and rectal epithelial damage caused by hyperosmolar lubricants may increase transmission rates of STIs, a suspicion supported by a small body of research.^1^^,^^2^^,^^49^

Lubricants containing highly osmolar glycerin have also been linked to bacterial vaginosis and changes in the vaginal flora.^47^^,^^50^ “A rise in vaginal pH typically indicates an overgrowth of gram-negative bacteria,” says Dezzutti. “Normally you have lactobacilli, but instead [with this overgrowth] you find *E. coli* and *Gardnerella*. It’s similar to the effects of using antibiotics.” However, another study found no obvious damage to the vaginal flora of rhesus monkeys from the use of K-Y Warming gel, despite the product’s high glycerin content.^51^

Cone has reported evidence that glycerin, glycerol monolaurate, polyethylene glycol, and propylene glycol—all used as excipients, or bulking agents, in lubricants—increased the transmission of genital herpes infections in the mouse vagina.^2^ Cone and colleagues wrote, “Although excipients are often called ‘inactive ingredients’ and are widely considered to be benign, these ingredients do have activities and toxicities.”^2^ They further wrote that none of the excipients used in personal lubricants or other vaginal products have been tested specifically to see if they increase susceptibility to STIs via mucous membranes.^2^

## Varied Regulation

The FDA regulates feminine hygiene products in three different ways. Tampons, sanitary pads, and most personal lubricants are considered medical devices,^52^ while medicated douches, anti-itch creams, and certain yeast infection treatments are regulated as over-the-counter drugs.^53^ In the third category, deodorizing sprays, powders, washes, nonmedicated douches, and most wipes are considered cosmetics, which must not contain any “poisonous or deleterious substance which may render it injurious to users under the conditions of use prescribed in the labeling,” according to FDA regulations.^54^

Products classified as medical devices need not disclose ingredients on packaging, which has led WVE to petition tampon manufacturers to start listing this information. Some feminine products are labeled “for external use only,” which can be confusing for consumers. Jamie McConnell, WVE’s policy director, explains that the instructions for one moisturizing gel say “apply a small amount of gel to the vaginal opening” but on the product label, it says “for external use only.” Yet, she says, “If you’re using it on your vagina, there’s going to be some internal exposure.”

A feminine wash containing color additives approved for external use only led WVE to contact the FDA for clarification, given that contact with mucous membranes seems inevitable during washing. Beth Meyers, a spokeswoman for the FDA Office of Cosmetics and Colors, says, “The term ‘external use’ as applied to color additives specifically excludes use on any mucous membrane.” Meyers adds, however, “Generally speaking, an isolated report or unconfirmed anecdotal information does not constitute adequate support for enforcement action.”

With greater awareness of the chemicals in feminine hygiene products, the unique characteristics of the vaginal region, and the potential for health disparities among various groups of women through culturally determined use of these products, it is apparent that more studies are needed to connect the dots.

“The big data gap is what are the adverse health effects [if any] arising from the chemical exposures from these feminine hygiene products and to what extent have we been underestimating exposures because we haven’t been accounting for this unique exposure route and the potential for the differential [absorption],” says Zota. “It’s now a call to the environmental health community to address some of these gaps.”
